# The Role of Eosinophils, Eosinophil-Related Cytokines and AI in Predicting Immunotherapy Efficacy in NSCLC

**DOI:** 10.3390/biom15040491

**Published:** 2025-03-27

**Authors:** Fausto Omero, Desirèe Speranza, Giuseppe Murdaca, Mariacarmela Cavaleri, Mariapia Marafioti, Vincenzo Cianci, Massimiliano Berretta, Marco Casciaro, Sebastiano Gangemi, Mariacarmela Santarpia

**Affiliations:** 1 Medical Oncology Unit, Department of Human Pathology “G. Barresi”, University of Messina, 98125 Messina, Italy; faustoomero@hotmail.it (F.O.); desiree.speranza@gmail.com (D.S.); mariacarmelacavaleri@libero.it (M.C.); marafiotimariapia@gmail.com (M.M.); mariacarmela.santarpia@unime.it (M.S.); 2 Department of Internal Medicine, University of Genoa, 16132 Genoa, Italy; 3 Department of Biomedical and Dental Sciences and Morphofunctional Imaging, Section of Legal Medicine, University of Messina, Via Consolare Valeria, 1, 98125 Messina, Italy; enzocianci.1997@gmail.com; 4 Medical Oncology Unit, Department of Clinical and Experimental Medicine, University of Messina, Via Consolare Valeria, 98125 Messina, Italy; massimiliano.berretta@unime.it; 5 School and Operative Unit of Allergy and Clinical Immunology, Department of Clinical and Experimental Medicine, University of Messina, 98125 Messina, Italy; marco.casciaro@unime.it (M.C.); gangemis@unime.it (S.G.)

**Keywords:** immunotherapy, NSCLC, lung cancer, biomarkers, eosinophils, cytokines, IL-31, IL-33, artificial intelligence

## Abstract

Immunotherapy and chemoimmunotherapy are standard treatments for non-oncogene-addicted advanced non-small cell lung cancer (NSCLC). Currently, a limited number of biomarkers, including programmed death-ligand 1 (PD-L1) expression, microsatellite instability (MSI), and tumor mutational burden (TMB), are used in clinical practice to predict benefits from immune checkpoint inhibitors (ICIs). It is therefore necessary to search for novel biomarkers that could be helpful to identify patients who respond to immunotherapy. In this context, research efforts are focusing on different cells and mechanisms involved in anti-tumor immune response. Herein, we provide un updated literature review on the role of eosinophils in cancer development and immune response, and the functions of some cytokines, including IL-31 and IL-33, in eosinophil activation. We discuss available data demonstrating a correlation between eosinophils and clinical outcomes of ICIs in lung cancer. In this context, we underscore the role of absolute eosinophil count (AEC) and tumor-associated tissue eosinophilia (TATE) as promising biomarkers able to predict the efficacy and toxicities from immunotherapy. The role of eosinophils and cytokines in NSCLC, treated with ICIs, is not yet fully understood, and further research may be crucial to determine their role as biomarkers of response. Artificial intelligence, through the analysis of big data, could be exploited in the future to elucidate the role of eosinophils and cytokines in lung cancer.

## 1. Introduction

Several clinical trials have shown improved overall survival (OS) and progression-free survival (PFS) in patients with advanced NSCLC treated with first-line chemo-immunotherapy or immunotherapy alone [1,2,3].

Considering the positive survival outcomes observed in these trials, first-line immunotherapy, with or without chemotherapy, has become the treatment of choice for non-oncogene-addicted advanced NSCLC [4,5,6,7].

ICIs are monoclonal antibodies that link and inhibit immune checkpoints. This inhibition causes the activation of immune cells, particularly lymphocytes, against the tumor [8,9].

The most relevant immune checkpoints are programmed death 1 (PD-1), PD-L1, and cytotoxic T lymphocyte-associated protein 4 (CTLA-4). CTLA-4 primarily operates in lymphatic tissues, while PD-1 and PD-L1/PD-L2 are crucial components in peripheral tissues [10,11,12,13].

Antigen-presenting cells (APCs) express surface ligands that bind CTLA-4 which, together with CD28, inhibit the T-cell response. Similarly, PD-L1 and PD-L2, expressed on inflammatory cells and some of the tumor microenvironment cells, bind to PD-1, which inhibits different immune cells like T lymphocytes, B lymphocytes and natural killer (NK) cells [14,15,16,17].

Antigen presentation, co-stimulatory signals, and cytokine expression are necessary to effectively activate T-cells, and many other signals are essential to prevent self-cell attacks [14,18].

Anti-PD-1/PD-L1 antibodies demonstrate their antitumor effects by inhibiting the interaction between PD-L1 and its receptor, PD-1, thereby activating cytotoxic T lymphocytes to attack and kill tumor cells [19,20,21].

PD-1 has another ligand, PD-L2, which has been investigated in NSCLC treated with immunotherapy. Studies have confirmed that PD-L2 could also be a target of ICIs in lung cancer that do not express PD-L1 [21,22,23,24,25,26,27].

So far, tissue programmed cell death ligand 1 (PD-L1) expression has been validated as a predictive biomarker of ICI treatment outcomes, including anti-PD-1/PD-L1 therapy, in NSCLC [28,29].

Previous studies have shown that patients who experienced immune-related adverse events (irAEs) had a higher Overall Response Rate (ORR) and a longer Progression-Free Survival (PFS) than those who did not [9,30,31,32].

Nevertheless, some patients do not benefit from ICI use or experience different grades of immune-related adverse events (irAEs), and several studies have sought to identify other biomarkers of response to ICI treatment, in addition to PD-L1 [33,34,35,36,37].

During the past few years, the application of artificial intelligence (AI) in medicine has increased significantly, offering novel approaches to diagnosis, prognosis, and treatment selection. AI-driven models, particularly those based on machine learning and deep learning, have shown promise in predicting treatment responses, identifying novel biomarkers, and optimizing personalized therapeutic strategies. In the field of cancer, AI has been applied to analyze imaging, histopathological data, and multi-omics datasets, providing insights into tumor microenvironment interactions and immune responses. For these reasons, we inserted an AI section to speculate whether artificial intelligence could help in identifying emerging biomarkers and optimize patient selection, contributing to more personalized and effective medicine.

We performed an updated literature search on immunotherapy, NSCLC, and eosinophils, on the main medical research databases (PubMed, Scopus, and Web of Science). The following search terms were used: immunotherapy, NSCLC, lung cancer, biomarkers, eosinophils, cytokines and artificial intelligence. Duplicate articles were removed and only articles in English were considered.

## 2. Biology of Eosinophils

**Figure 1:**
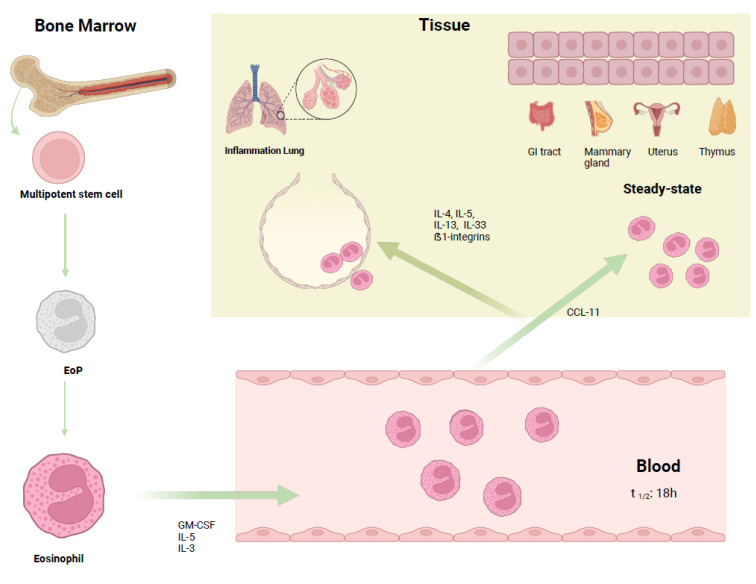
Main transcription factors in the eosinophil lineage: The eosinophil lineage commitment is driven by the transcription factors c/EBPα, GATA-1&2, FOG, PU.1, TRIB-1 and IRF8. Mature eosinophils are further stimulated by cytokines like IL-5 and IL-33.

Granulocytes are a heterogeneous group of blood cells comprising neutrophils, eosinophils, and basophils, playing a crucial role during inflammation and infections. Eosinophils activity is mainly directed against parasitic infections, but they also take part in several pathological processes linked to allergic phenomena (e.g., asthma and rhinitis) as well as non-allergic diseases (e.g., chronic spontaneous urticaria) [38]. These originate from multipotent bone marrow progenitors [39] (Figure 1).

Once activated, the progenitor cell undergoes differentiation into a mature cell after intermediate steps. Although the maturation mechanism has not been yet completely elucidated, some stimulating factors, like IRF8, c/EBP, GATA-1, GATA-2, have been described [40].

Differentiation is also characterized by the decrease in the amount of some proteins normally highly expressed at the initial stages, including FOG-1, by hematopoietic progenitor cells (HPCs), while some others, including GATA-1/2, increase in amount during the former’s transition into eosinophilic progenitor (EoP) cells [41].

Cytokines are also important for the differentiation of progenitors particularly IL-3, GM-CSF and IL-5; the first two are proteins also involved in the maturation of other myeloid cells, while IL-5 is more specific to the line of eosinophils. Indeed, in mouse models, IL-5 overexpression was associated with eosinophilia [42,43]. IL-18 can induce eosinophilia and induce eosinophil differentiation [44].

The cytoplasm of mature eosinophils is rich in granules containing various proteins involved in infectious and allergic processes. The main proteins are major basic protein (MBP), eosinophil peroxidase (EPO), eosinophil cationic protein (ECP) and eosinophil-derived neurotoxin (EDN). The MBP is fundamental for the immune response against helminth infections; the same protein is then responsible for mucosal damage in allergic response [45].

The eosinophil cationic protein takes part in antiviral activity, the synthesis of immunoglobulins by B lymphocytes and the degranulation of mast cells [46], while peroxidase, through different chemical mediators, increases cellular oxidative stress, thus causing apoptosis and necrosis phenomena [47].

In terms of physiological status, eosinophils are predominantly present in the gastrointestinal tract, spleen, lymph nodes, thymus, mammary glands and uterus and have a short half-life. Eosinophils migrate to these sites under the stimulus of chemotactic agents like eotaxin-1, RANTES and MIP-1, and take part in processes such as maturation of the uterus and mammary gland [48,49].

Interleukin-5 is an essential factor in both the maturation of the progenitor eosinophilic cell and in the activity of this granulocyte during parasitic infections and in response to allergens, together with other factors such as interleukins 4 and 13 [50]. Its function can be mediated by the activation of its receptor (IL-5Rα) or by stimulating the secretion of other mediators [51].

Among the various cytokines and growth factors involved in eosinophil biology, IL-33 is a significant player. This cytokine, which is derived from epithelial and tumor cells and belongs to the IL-1 cytokine family, is an essential alarmin in the body’s defense against tumors. IL-33 not only regulates tumor growth but also helps to prevent metastatization [52,53].

## 3. Role of IL-33 and IL-31

As outlined above, cytokines are fundamental in the development and activity of both eosinophils and other immune cells. In particular, the activity of IL-31 and IL-33 seems to be crucial in the formation of an immune response against cancers.

IL-33 interacts with the interleukin receptor-like 1 (IL-1RL1) protein and ST2. This interaction influences Th2 cells, type 2 innate lymphoid cells (ILC2s), mast cells and granulocytes, favoring IL-4, IL-5, and IL-13 release. These cytokines are involved in allergic inflammation [54,55].

Numerous authors have demonstrated that IL-33 activates various immune cells, including CD8+ lymphocytes, NK cells, regulatory T-cells (Tregs), B cells, neutrophils, macrophages and dendritic cells (DCs) [56].

IL-33’s role in cancer development is controversial. In *IL33−/−* and *IL1rl1−/−* mice, tumor growth was faster than in the control. Furthermore, the administration of antibodies that block IL-33 has been demonstrated to have protumoral effects. On the other hand, the exogenous administration of IL-33 and its overexpression is associated with better immune responses to tumors [57,58].

Pro-tumor effects may derive from the suppression of the T-cell (Treg) population, a subpopulation of T-cells that promote tumor immune escape and from the polarization of tumor-associated macrophages (TAMs) to M2, which promote tumor growth, angiogenesis and, through metalloproteinase release, metastatization [59,60].

There are multiple mechanisms that lead to antitumor immune responses mediated by IL-33. For example, IL-33-activates eosinophils that release more effector molecules, through the CD11b/CD18 link, essential for cancer cell death [61].

IL-33 can also increase immune responses to cancer cells reducing Treg expansion or enhancing tumor-infiltrating ILC2s. Antitumor response is also supported by cDC1s. In cancer models, CD103+ DCs, activated by IL-33, stimulate CD8+ T-cell activity, leading to anticancer responses [62].

Basophils, activated by IL-33, increase the release of several degranulating molecules like CD63 and granzyme-B, leading to tumor cell killing in vitro [63]. It was also hypothesized that IL-31 and IL-33 were linked to vitamin D levels [64]. Interestingly, the intranasal use of IL-33 attracts eosinophils into the lungs, helping to prevent pulmonary metastases [65].

Based on these studies, IL-33 injection could be proposed in order to generate highly immunogenic cDC1s. In addition, IL-33 derived factors stimulate highly immunogenic human monocyte (hMo-DCs) growth [66].

As reported above, IL-33 has a dual role (pro- or anti-tumoral) depending on the conditions. Its polar role depends on its ability to modulate specific cellular populations within the tumor microenvironment. For example, while this alarmin enhances anti-tumoral responses by activating CD8+ lymphocytes and eosinophils, in other contexts, it can favor macrophage polarization toward the M2 phenotype, which supports angiogenesis and tumor progression.

IL-31 is a cytokine, that is IL-4-dependent, produced by T-cells such as CD4+ [67,68].

Through the activation of three main intracellular pathways, JAK/STAT, PI3K/AKT and MAPK [69], IL-31 regulates several biological functions, such as inflammation, cell proliferation, and tissue remodeling [70].

The precise role of IL-31 in tumor biology and treatment response remains uncertain. Heterodimeric receptor IL-31RA/OSMRβ is expressed both on numerous immune cells, like monocytes, macrophages, and DCs, and on non-immune cells, like different epithelial cells [71].

IL- 31/IL-31RA was firstly studied in cutaneous T-cell lymphoma (CTCL), where IL-31 was correlated to staging. IL-31RA mRNA can be found in melanoma, glioblastoma, osteosarcoma, and myelomonocytic leukemia cell lines, as well as in CTCL [72,73,74].

The intraperitoneal injection of IL-31 in a murine breast cancer model resulted in CD8+ T-cell infiltration, which led to an immune response against tumor cells; better survival was also demonstrated due to the modulation of the release of cytokines like IL-10 and IL-2. IL-31RA mRNA expression is closely connected to IL-2/IL-4 mRNA expression, granzyme B protein expression, and improvements in the overall survival of BC patients [71].

IL-31 is also correlated with disease progression and cutaneous toxicities to EGFR-directed TKIs [75].

IL-31 is considerably reduced in gastric cancer. It also seems that IL-31 has antitumor effects in colon cancer, as MC38 cells that were silenced by IL-31 showed faster tumor growth in C57Bl/6 mice than IL-31-expressing control cells [76,77].

Higher IL-31 levels in patients with endometrial cancer than in controls were reported and were also correlated with immune cell infiltration and p-STAT3 expression in ovarian cancer, affecting the prognosis of patients [78,79]. Notably, IL-31 and IL-33 levels were correlated with clinical characteristics like staging, invasion, and metastasis. Furthermore, their sensitivity and specificity surpassed traditional tumor markers (e.g., CEA, CA-125, CA19-9), particularly when combined with clinical parameters [78].

IL-31-expressing breast tumors showed increased cytotoxic T-cell activity and decreased levels of CD4+, MDSCs, and macrophages. This immune shift is linked to a cytokine profile that supports antitumor immunity. The authors demonstrated that IL-31 reduces CD4+ T-cell proliferation and Th2 factor expression, while boosting CD8+ T-cell activation by inhibiting MDSC function [71].

Based on the above data, it remains crucial to better elucidate the roles of IL-31 and IL-33 and the related pathways in different tumors, as well as determine how they can be exploited as biomarkers for clinical use [80].

## 4. Role of Eosinophils in Cancer

**Figure 2:**
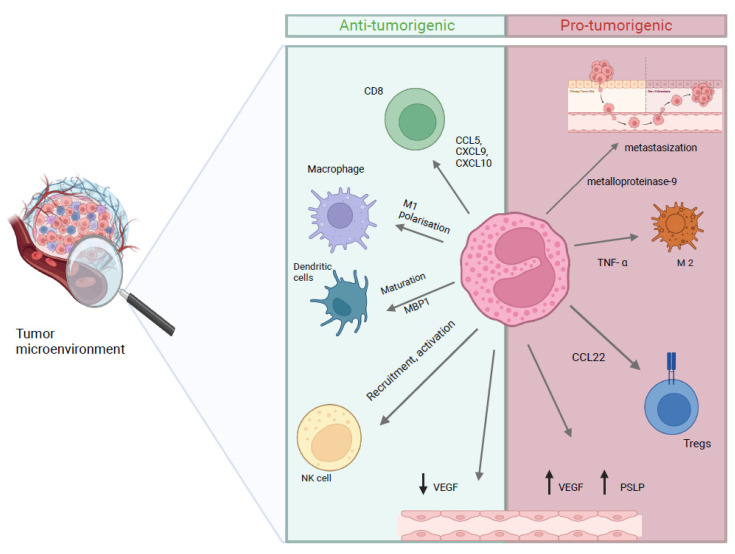
Mechanisms underlying the anti-tumorigenic and pro-tumorigenic roles of eosinophils. Eosinophils in the TME can promote tumor growth by inducing angiogenesis, releasing metalloproteinase-9 and driving M2 macrophage polarization through TNF-α. On the other hand, eosinophils play an anti-tumor role through the M1 polarization of macrophages, the recruitment and activation of immune cells such as NK cells, DC and CD8, and reduced VEGF expression.

Eosinophils demonstrate a dual role in tumor dynamics, displaying both anti-tumor and pro-tumorigenic activities through various mechanisms. Mechanisms are various and may include cell-mediated response, like T-cell recruitment and chemokine release, DC stimulation, vascular modification, and enhancement of intra-tumor surveillance, thereby exerting direct anticancer effects [81,82,83,84,85,86] (Figure 2).

For this reason, the migration of eosinophils to tumor tissues depends on the latter’s ability to cause an inflammatory reaction by inducing necrosis and stimulating the release of additional chemoattractant factors from immune cells [86].

Among the chemotactic factors that mediate this migration are IL-5 and IL-3 and eotassin (CCL11). Eosinophils interact with cancer cells both directly and indirectly through contact with other TME cells. Different studies have shown contrasting results with regards to the role of eosinophils in cancer, as they show both immunostimulant activity, as in melanoma models, and immunosuppressive activity [85].

Eosinophils are recruited in the tumor microenvironment by several cytokines (IL-4, -5, -13 and eotaxin), as shown in in vitro lung cancer models [87].

They can increase the infiltration of various immune cells, like CD8+ T lymphocytes, which directly attack cancer cells. An increase in gamma interferon release (IFNγ) has been observed in several models. IFNγ activation of eosinophils may lead to the transformation of macrophages into M1, which would appear to have anti-tumor activity [88].

In addition, IFNγ-activated eosinophils can release mediators, such as oxygen-reactive species, which cause damage to cancer cell DNA [89].

Eosinophils can also interfere with angiogenesis, a crucial process by which the tumor, forming new vessels, is able to grow, progress and develop metastases [83].

About 28% of prostate cancer patients, treated with immunotherapy, showing a better response to therapy with OS prolongation, had an increased AEC [90].

Caliman et al. stated that eosinophils could be predictive biomarkers for treatment efficacy and toxicity, offering a means to personalize immunotherapy by identifying patients likely to benefit most [91].

IL-2 injection, as an immunotherapy, has been associated with eosinophilia. IL-2 is able to increase the activity of eosinophils and can partly explain their anticancer activity [92].

In several experiments, through cytokine manipulation, the increase in eosinophils in the TME was associated with a significant tumor size decrease [82].

However, data from the literature, mostly from in vitro studies, indicate that eosinophilic infiltration within the tumor microenvironment can also stimulate tumor growth.

For example, oral cell carcinoma showed a size reduction in the case of a low AEC. The absence of CCL3, associated with eosinophil deficiency, seems to negatively affect angiogenic activity and the release of metalloproteinases, which increase tumor invasion and increase the risk of metastases. The factors involved include TGF-β 1, VEGFa and VEGFb [82,93].

Some cytokines released by tumor cells can activate eosinophils, causing an increase in cervical carcinoma. The increased secretion of CCL17, induced by hypoxic conditions, attracted more eosinophils in the TME and led to a high level of immunosuppressant cytokines. Tumor-generated thymic stromal lymphopoietin is also involved in angiogenesis [94].

IL-33-dependent lymphoid cells, present in lung cancer environment, also cause a reduction in the production of interferon and a suppression of cell-mediated immune activity through interference with the function of NK cells. This complex mechanism is also dependent on the eosinophilia induced by IL-5 [95,96].

Eosinophils also can result in macrophage polarization in M2, the immunosuppressive subtype, via the secretion of intermediate cytokines. By inhibiting the MAPK pathway and stimulating anti-inflammatory activity, they can cause the transition of M1 macrophages into M2 ones [97,98].

IL-31, a pro-inflammatory cytokine, is involved in tissue remodeling and may indirectly support tumor progression by enhancing eosinophil-mediated inflammation [99]. IL-33 enhances eosinophil-mediated tumor cell killing by promoting adhesion and degranulation, which is crucial for their cytotoxic function in the TME [53].

This alarmin recruits eosinophils to the TME and activates them to exert cytotoxic effects on tumor cells. This cytokine upregulates adhesion molecules and degranulation markers, facilitating eosinophil–tumor cell interactions. On the other hand, IL-31’s role in the TME is less direct; its involvement in inflammatory pathways suggests that it may modulate eosinophil activity and influence their pro-tumorigenic functions.

These contrasting data on the activity of eosinophils in tumors probably reflect their plasticity. It is likely that the activity of eosinophils, as for other cells in the immune system, is not unique, but rather affected by multiple factors [100,101].

## 5. Eosinophils in NSCLC

Despite increasing evidence supporting the effectiveness of anti-PD1 and PD-L1 therapies in treating various cancers, including NSLC, there is a significant lack of reliable biomarkers to predict patient responses and potential toxicity from these immunotherapeutic agents in routine clinical practice [102,103].

The reactivation of T lymphocytes mediated by the activity of ICIs leads to an inflammatory response [104].

Eosinophils show both stimulating and inhibitory activity on the immune system which varies according to cancer type and the surrounding microenvironment [105,106].

Direct anti-tumor effects can be achieved using cytokines and improved immune surveillance, like T-cell enrolment and polarization [81,85].

Some authors have highlighted that eosinophils are present in small quantities in tissue samples, suggesting some limitations to studying their function with current techniques. These data explain why eosinophils have so far been studied only to a limited extent in the context of lung cancer. However, technological advances may soon fill this gap through studies on mouse models [84,107,108].

Peripheral blood biomarkers are more appealing than tumor tissue for the routine monitoring of responses during immunotherapy, primarily because they are easily available and can be collected at different time points during therapy [37].

AEC is an easily accessible parameter because it is commonly included in the complete blood count that is requested before and during immunotherapy. An increase in eosinophil count can be linked to various pathological conditions like allergic reactions, inflammatory disorders, parasitic infections, viral diseases and neoplastic diseases [109,110].

Ye and colleagues studied the role of eosinophil peroxidase (EPO) expression in 90 lung adenocarcinoma samples. They investigated the relationship between EPO, some of the clinical characteristics of patients, and disease prognosis in lung cancer patients. EPO mRNA expression was higher in tumor samples than in healthy lung tissue. High EPO expression also correlated with disease stage. Patients who showed poorer survival also had high levels of EPO [111].

Tataroglu et al. published a study on the association between mast cells (MCs), TAMs, eosinophils, tumor vasculature and TNM stage in NSCLC samples. There was not any significant correlation between tumor’s staging, TAMs and eosinophil counts. The lack of correlation is discordant with the results of some previous studies on NSCLC and other tumors, perhaps due to the heterogeneity in the methodologies used [112].

### 5.1. Eosinophils as Predictive Biomarkers of Clinical Efficacy from Immunotherapy

There have been few studies that have examined whether AEC can predict clinical outcomes and irAE development in NSCLC treated with immunotherapy [113,114].

The association between peripheral eosinophil count and the therapeutic effect of ICIs was investigated both in solid tumors like melanoma and lung cancer [113], and in hematological malignancies like Hodgkin lymphoma [115].

NSCLC cells produce several factors, including IL-5, which have a crucial role in the growth, differentiation and activation of eosinophils, which may explain an increase in eosinophils in the blood and tumor tissue under these conditions [111,116].

This result may be due to the release of molecules that lead to an increase in granulocytopoiesis, as demonstrated in mouse models [117,118].

The presence of eosinophils both in peripheral blood and tumor tissue (tumor-associated tissue eosinophilia) has been identified as a prognostic indicator of improved clinical outcomes during immunotherapy [119].

Huai et al. have analyzed a cohort of 189 patients treated with neoadjuvant immunotherapy. The authors have evaluated the eosinophilic fraction before and after neoadjuvant treatment. They found that higher levels after neoadjuvant treatment correlated with major pathological responses, without any correlations with OS or EFS [120].

A retrospective analysis of the research by Takeuchi et al. focused on the peripheral eosinophil count of 166 patients with NSCLC treated with ICIs. Patients were stratified in threes group according to the levels of eosinophils: <100 cells/μL, 100–500 cells/μL, >500 cells/μL. The mOS of the first group was 339 days, that of the second was 667 days, and it was 143 days for patients with more than 500 eosinophils per μL, with a statistically significant difference [121].

Other authors have evaluated the variability in the relative eosinophil count before immunotherapy and four weeks after the start of ICI treatment in a cohort of 151 lung cancer patients. The primary endpoint was the disease control rates (DCRs), which were higher in patients with increased eosinophil counts at 4 weeks than in those without increased counts. Overall survival was also different in the two groups, with a median survival of 674 days in the first group and 234 days in the second. The multivariate analysis confirmed these results [122].

Although in some studies the presence of an eosinophil count above the threshold of 500 cells/μL does not seem to show an increase in the clinical benefits of patients undergoing immunotherapy, in a group of 121 NSCLC patients, an eosinophil count above this threshold correlated with a statistically significant benefit in terms of OS (26.6 vs. 9.5 months) and PFS (13.8 vs. 4.6 months). At the same time, eosinophilia has also been linked to an increased likelihood of adverse events [123].

According to Tanizaki et al., eosinophil count may have predictive value in patients with NSCLC treated with nivolumab: a high AEC (≥150/μL), high lymphocyte levels (≥1000/μL), and low neutrophil levels (<7500/μL) at the start of nivolumab treatment have been significantly associated with better PFS and OS [113].

Sibille et al. evaluated the baseline and immunotherapy levels of white blood cells in patients with NSCLC. The AEC was found to increase during treatment. The high AEC observed (>0.15 cells/mL), in association with low neutrophil levels (<7.5 cells/mL) and high lymphocyte levels (>1.0 cells/mL), was correlated with high survival rates. On the other hand, very high levels of eosinophils (AEC > 0.5 cells/mL) were found to be associated with a higher frequency of irAEs. These clinical findings are explained by the significant immune activation occurring [124].

In the literature, there are other cases of lung adenocarcinoma treated with ICIs with subsequent hypereosinophilia and with favorable prognosis [34,125].

In a cohort of 25 patients with solid tumors, treated with IL-2 and lymphokine-activated killer cells, 11 patients had objective tumor regression, and the tumors involved were melanoma, colorectal cancer, renal cell cancer and lung adenocarcinoma [126].

Van Haelst Pisani et al. also demonstrated that the increase in eosinophil counts was a consequence of IL-2 infusion [127]. From these initial studies, subsequent data demonstrated a potential predictive role of eosinophils in clinical outcomes of ICIs in multiple types of cancer [128,129,130,131,132].

Eosinophils in the pleural exudate of patients with lung cancer may also be related to increased survival, with an OS of 766 days vs. 252 days for patients without eosinophilic pleural effusion [133].

Many other interesting data can be derived from the study of patients with other solid tumors who are undergoing immunotherapy.

In a meta-analysis conducted by Hu et al. [134], the presence of TATE was linked to improved overall survival among patients with various solid tumors. No significant association was found between TATE and disease-free survival. Furthermore, TATE demonstrated an inverse correlation with lymph node metastasis and lymphatic invasion.

Eosinophils exhibit anti-tumoral activity in various types of malignancies, like melanoma and gastric cancer. However, in other tumors, like cervical carcinoma and Hodgkin’s lymphoma, they correlated with poorer prognosis [119,135,136].

A hypereosinophilia associated with ICI therapy is occasionally reported. There are currently few reports of hypereosinophilia caused by nivolumab in the literature [137,138].

Some authors have hypothesized that determining the AEC a few weeks after the start of immunotherapy could provide important information on the efficacy of the therapy [132].

Some authors advocate for the existence of an eosinophilic syndrome driven by ICIs [108,124].

In their observational study, Scanvion et al. considered 37 cases of eosinophilia induced by ICIs. The median time to the AEC peak was 15 weeks. The median AEC was 2.7 g/L. In 21 out of the 37 cases (57%), eosinophil-related manifestations were reported. Eosinophilia remission was achieved in certain patients who were not treated with corticosteroids, whether they discontinued or continued on the ICIs [139].

### 5.2. Eosinophils as a Biomarker of Immune-Related Adverse Events (irAEs)

Recent evidence suggests that eosinophil count, in addition to being a potential biomarker of response, could be associated with irAEs in various tumors [114,140,141].

Regarding NSCLC, Chu et al. analyzed 300 patients with advanced NSCLC who received ICIs. Overall, 18% of them experienced ICI-related pneumonitis. This subgroup had high baseline AECs (27.7% if AEC ≥ 0.125 cells/mL vs. 9.8% if AEC < 0.125 cells/mL). However, patients with higher AECs had a better ORR (40.9% versus 28.8%) and longer PFS (8.93 months versus 5.87 months) [114].

Hu et al. conducted a retrospective analysis of NSCLC patients treated with ICIs in order to examine the connection between baseline characteristics and AEs. The most common reported irAEs were pneumonia, endocrine dysfunctions, myocarditis, and rash. In the multivariate analysis, an eosinophil percentage ≥ 1.15% combined with IFN-γ ≥ 3.75 ng/L were considered predictive factors of developing irAEs. About 55% of patients developed AES. Interestingly, patients with low-grade irAEs (grade 1–2) showed longer PFS compared to those with a high grade and those that had no irAEs [142].

In another study, baseline eosinophil count (high or low, with a cut-off value of 130 cells/μL) was correlated with irAEs development in a large population of advanced NSCLC patients receiving ICIs as a first- or second-line monotherapy. Overall, 43.7% patients developed irAEs, of which 18.9% were dermatological, 13.3% were endocrine, 10.1% were hepatic, 8.2% were gastro-intestinal, and 7.6% were pulmonary events. The high-AEC group reported an increased irAE risk [91].

Higher incidence of irAEs was also found in a patient with gastro-intestinal, urogenital, and cutaneous tumors treated with immunotherapy and exhibiting a high AEC at baseline (>0.045 × 10^9^/L) [143].

In a retrospective analysis, Pozorski et al. proposed neutrophil-to-eosinophil ratios (NERs) as a predictive indicator of the response to immunotherapy in patients with melanoma. The NER was calculated by dividing the absolute neutrophil count (ANC) by the absolute eosinophil count. In total, 223 patients were enrolled. Fifty-one cases of grade 3–4 irAEs were described. The baseline NER was not associated with irAEs; nevertheless, the multivariate test showed that at 1 month, it was associated with a lower risk of high-grade adverse events, paving the way for more research to investigate NERs in clinical settings [144].

Okano et al. described a case of a middle-aged man affected by oral melanoma with metastatic nodes. The patient started immunotherapy with nivolumab as a second-line therapy. During ICI treatment, the patient developed general weakness, and laboratory tests showed eosinophilia (21.1%) and hyponatremia. A complete hormonal examination demonstrated low ACTH and cortisol levels, while FSH and LH were normal. It was suspected that the patient had immunotherapy-induced hypophysitis. After undergoing hydrocortisone therapy, the patient’s eosinophil count decreased to 0.32%, and the clinical conditions improved, suggesting that eosinophilia, in some cases, may identify patients who can develop endocrine toxicity [145].

A retrospective single-center study evaluated the onset of hypopituitarism in a group of renal carcinomas treated with ICIs. A single patient developed adrenocorticotropic hormone deficiency secondary to hypopituitarism. Baseline characteristics showed an increasing eosinophil count before irAEs development. Four other subjects presented hypereosinophilia before the onset of the adverse event. The authors hypothesized a link between the development of hypereosinophilia and irAEs [146].

Several other researchers have described a relationship between eosinophilia and irAEs onset [139,147].

### 5.3. Limitations in NSCLC

The interplay between IL-31, IL-33, eosinophils, and the tumor microenvironment (TME) could significantly influence immunotherapy outcomes in NSCLC, and understanding these mechanisms is crucial for enhancing therapeutic efficacy and overcoming treatment resistance. IL-31 is implicated in promoting eosinophil recruitment and may contribute to an immunosuppressive TME, potentially hindering the effectiveness of immune checkpoint inhibitors (ICIs) [148], while IL-33 has been shown to reprogram the TME by enhancing T-cell responses when combined with anti-PD-L1 therapy; conversely, blocking IL-33 signaling can reduce immunosuppressive factors, thereby improving antitumor efficacy [66]. Eosinophils themselves modulate immune responses within the TME and often contribute to a pro-tumor environment, with their presence correlating with poorer outcomes in patients receiving ICIs [148]. Additionally, the heterogeneity of the TME, particularly between lung adenocarcinoma and squamous cell carcinoma—where the former exhibits a more active immune microenvironment compared to that of the latter, which is often more immunosuppressive—further affects immunotherapy responses [149]. Thus, while IL-31 and IL-33 may promote immune evasion under certain conditions, targeting these pathways could also pave the way for novel therapeutic strategies that enhance the efficacy of existing immunotherapies, reflecting a dual role in both resistance and treatment enhancement. The integration of studies focusing on IL-31/IL-33 signaling pathways, eosinophil function, and TME dynamics could provide novel insights and lead to improved therapeutic strategies in NSCLC.

Moreover, while emerging evidence supports the potential of eosinophils, IL-31, and IL-33 as biomarkers for predicting immunotherapy outcomes, there is a notable deficiency in studies that define quantitative thresholds. This is largely due to the heterogeneity of the tumor microenvironment and the dynamic nature of these biomarkers in different patient populations. The establishment of predictive thresholds will require standardized methodologies, including larger, multicentric clinical studies and integrated analyses that consider both biological and technical variabilities. This represents an important avenue for future research.

In the next paragraph, we propose the use of modern tools for spanning novel paths.

## 6. Perspectives: Artificial Intelligence and Biomarkers of Immunotherapy

Data regarding the role of eosinophils during ICI therapy in NSCLC are conflicting. Moreover, it is still difficult to define a threshold value for eosinophils and cytokines that can predict the efficacy of ICIs. Through different pathways, eosinophils appear to have both stimulating and suppressive activity on lung cancer cells. Molecular advances may improve scientific data, allowing us to better understand eosinophil plasticity.

Innovative analysis of lung cancer biopsy or cytology, focused on the TME and immune cells, may help us to better understand how these interact with the tumor and vary according to immunological therapy.

It might be interesting to carry out a comparative study in populations with higher or lower tumor eosinophilic infiltrates.

New technologies like artificial intelligence (AI), through the analysis of large data, could make it possible to better understand the role of eosinophils in NSCLC oncogenesis, its treatment, and the complex network of molecular and cytokine pathways underlying this interaction. AI is driving precision medicine in oncology. By analyzing complex medical data, AI is enhancing the management of several malignancies. From accurate staging and prognosis assessment to personalized treatment plans and monitoring disease progression, artificial intelligence is empowering clinicians to make informed decisions and improve patient outcomes. In medicine, this new technology involves applying machine learning algorithms to medical data to enhance healthcare processes [150,151].

Machine learning is a method of teaching computers to learn from data, like humans learn from experience; it is divided in three main types: supervised learning, unsupervised learning (UL), and semi-supervised learning (SSL). Deep learning (DL) is a powerful subtype of ML that uses artificial neural networks [152]. This advanced technology has the potential to revolutionize biomarker discovery by analyzing and integrating vast amounts of multi-omics data, such as genomics, transcriptomics, epigenomics, radiomics, and pathomics [153].

Several studies have focused on AI’s involvement in the identification of immunotherapy biomarkers. A tumor microenvironment is a dynamic ecosystem surrounding a tumor, composed of various cells, including cancer cells, immune cells, and fibroblasts. It is involved in modulating the immune response to cancer cells, in some cases leading to an increase in lymphocyte activity toward the tumor and in others having an immunosuppressive effect [154]. Through the use of AI, it is possible to evaluate the morphological characteristics of cancer cells and the tumor microenvironment. In the future, it will be possible to analyze the immunological infiltrates within the tumor, including eosinophils, and their distribution in the tumor mass, so as to identify its correlation with the outcomes for patients, especially in response to immunotherapy [155].

For example, PD-L1 and TMB are response biomarkers in lung cancer treated with ICIs. The first, as already described, is a superficial protein that regulates the interaction with lymphocytes; the second, on the other hand, is a measure of somatic mutations presented by tumor cells and is directly related to neoantigen development. High levels of TMB therefore correlate with a better response to immunotherapy [155].

Research on PD-L1 is necessarily carried out today via biopsies but may suffer from several limitations. The ability of ML to analyze a large number of multi-omics data (demographic, pathological, laboratory) could reduce these limitations. ML has also been used in some complex PET/CT-based radiological algorithms to predict the levels of PD-L1 in tumors [156].

In some studies, artificial intelligence has been used to study TILs in NSCLC samples and their correlation with patient survival. This correlation was also associated with the intra-tumoral or stromal localization of TILs in the biopsy [157].

AI allows us to correlate the numerous demographic, pathological and laboratory data of patients affected by NSCLC, and we believe that in the future it can help to better clarify, through radiomic and pathological analysis, the role of eosinophils in their interaction with cancer cells and other immune cells to regulate the ICIs response. As described above, some cytokines are essential in the maturation of progenitor eosinophilic cells and in the activation of mature eosinophils, among them IL-31, IL-33, IL-4 and IL-5.

Wei F. et al. developed a predictive model, CIRI, to identify NSCLC patients who may benefit from immunotherapy. The model employed a machine learning approach to analyze changes in 93 circulating cytokine levels, including IL-33, at baseline and 6 weeks after the initiation of therapy. The CIRI model was found to be highly accurate in predicting patients’ outcomes, with a significant association between higher CIRI scores and worse overall survival [158].

While AI/ML technologies hold promising potential, it is imperative to balance technological advancements with human expertise. Rigorous validation, ethical considerations, and regulatory oversight are necessary to mitigate risks and ensure patient safety. Healthcare professionals must be equipped with the knowledge and skills to effectively utilize these tools and maintain a human-centered approach to patient care.

The limitations of artificial intelligence in biomarker research are numerous. Certainly, the first limit is the quality of the data for the creation of the analysis model: it is necessary to create a good predictive model that is based on a large amount of data that are also qualitatively accurate. Once the model is obtained, it cannot always be generalized; the generalization of data must take into account the variability of patients and numerous demographic and environmental forms of information. Lastly, the ethical and legal aspects of using AI in biomarker research are crucial: it is important to protect patients’ privacy, ensure proper access to health data and address the problem of informed consent [159].

## 7. Conclusions

Data regarding eosinophil involvement in lung cancer treated with ICIs are conflicting. However, clinical data suggest that elevated blood eosinophils could be a biomarker of improved outcome with ICIs.

The disparities between preclinical and clinical data are a key limitation.

Artificial intelligence can support and become fundamental in the prediction of the response to ICIs, particularly, in the analysis of the relationship with eosinophils, through the study of the TME, research on secreted cytokines and morphological analyses of tumors. AI can also help in uncover novel therapeutic targets for combination with currently approved therapies. This potential arises from the dual strengths of AI: accuracy and explainability. AI models are highly accurate in analyzing complex, high-dimensional datasets, such as genomic, proteomic, and clinical data, uncovering subtle patterns and correlations that traditional approaches might miss. This enables the identification of molecular pathways or proteins that influence both immune activation and tumor response, ensuring that the proposed targets are reliable and not false positives. Additionally, the explainability of AI models allows researchers to understand the biological mechanisms behind these predictions. For instance, if a cytokine is identified as a target, explainability tools can reveal its role in modulating immune responses, building confidence in its validity, and guiding experimental validation. More stringent clinical research and functional studies will allow for a more complete understanding of the role of eosinophils in NSCLC and of their potential for use as reliable biomarkers in clinical settings.

## Data Availability

Not applicable.
